# Constraining functional coactivation with a cluster-based structural connectivity network

**DOI:** 10.1162/netn_a_00242

**Published:** 2022-10-01

**Authors:** Inhan Kang, Matthew Galdo, Brandon M. Turner

**Affiliations:** Department of Psychology, Ohio State University, Columbus, OH, USA

**Keywords:** Structural and functional connectivity, Diffusion tensor imaging, fMRI, Gordon parcellation, Chinese restaurant process, Factor analysis

## Abstract

In this article, we propose a two-step pipeline to explore task-dependent functional coactivations of brain clusters with constraints from the structural connectivity network. In the first step, the pipeline employs a nonparametric Bayesian clustering method that can estimate the optimal number of clusters, cluster assignments of brain regions of interest (ROIs), and the strength of within- and between-cluster connections without any prior knowledge. In the second step, a factor analysis model is applied to functional data with factors defined as the obtained structural clusters and the factor structure informed by the structural network. The coactivations of ROIs and their clusters can be studied by correlations between factors, which can largely differ by ongoing cognitive task. We provide a simulation study to validate that the pipeline can recover the underlying structural and functional network. We also apply the proposed pipeline to empirical data to explore the structural network of ROIs obtained by the Gordon parcellation and study their functional coactivations across eight cognitive tasks and a resting-state condition.

## Author Summary

In this article, we propose a two-step pipeline to explore task-dependent functional coactivations of brain clusters with constraints imposed from structural connectivity networks. In the first step, the pipeline employs a nonparametric Bayesian clustering method that can estimate the optimal number of clusters, cluster assignments of brain regions of interest, and the strength of within- and between-cluster connections without any prior knowledge. In the second step, a factor analysis model is applied to functional data with factors defined as the obtained structural clusters and the factor structure informed by the structural network.

## INTRODUCTION

A crucial insight in modern neuroscience is that the interaction between brain structures can be just as important as the activity in the individual regions themselves (Catani et al., 2012). Interactions in the brain emerge through some type of connectivity, whether it be structural or functional, and the study of the connectivity relations is called “connectomics” (Sporns, Tononi, & Kötter, 2005). Whereas structural connectivity focuses on characterizing the degree to which white matter fiber bundles connect one brain region to another, functional connectivity characterizes the statistical relations between brain regions, such as sets of pairwise correlations. Another type of connectivity is called effective connectivity, where the structural and functional relations are often used to characterize how changes in the activity of one brain region are caused by changes in the activity of another (Friston, 2011; McIntosh, 2010).

One key observation is that structural connectivity can be used to place strong constraints when studying functional connectivity (Bullmore & Sporns, 2009). Indeed, causal relationships between regions of interest (ROI) could only occur in a biological system if some type of anatomical connection allows one region to alter the activity in another region. Although it is still possible for areas to show a functional relationship (e.g., a correlation) without a structural relationship, if there is a structural relationship, some functional dependency should emerge from it (Honey et al., 2009; Messé, Rudrauf, Giron, & Marrelec, 2015). Over time, considerable evidence has accrued suggesting that predictions about functional coactivation are improved when one has knowledge of an individual’s structural connectivity (Hagmann et al., 2008; Honey et al., 2009; Saygin et al., 2012; van den Heuvel, Mandl, Kahn, & Hulshoff Pol, 2009).

Of particular interest to us is relating structural brain clusters to functional activation of brain regions. Earlier studies in neuroscience have shown that brain areas construct structurally connected clusters through, for example, white matter tracts (Hinne, Ekman, Janssen, Heskes, & van Gerven, 2015; Mars et al., 2011; van den Heuvel & Sporns, 2019). Structural connections within and between clusters produce circuitry of neural activity flows, and thus, structural connectivity constraints on functional connectivity can be further improved by incorporating the information of clustered network communities. The key idea is to define functional clusters based on structural clusters and study coactivations of these clusters. A pattern of functional coactivations has considerable individual differences and it can also vary by behavioral task. This heterogeneity in the functional network can also be studied by changes in within- and between-cluster coactivations.

In this article, we attempt such an approach by using a type of nonparametric clustering to first analyze structural data, and use the resulting latent structure to inform a factor analytic model of functional coactivation. The notion of decomposing structural information about the brain into clustered communities is certainly not new (Allen et al., 2014; Anwander, Tittgemeyer, von Cramon, Friederici, & Knösche, 2007; Hansen, Battaglia, Spiegler, Deco, & Jirsa, 2015; Hinne et al., 2015; Mars et al., 2011). However, one originality of our approach comes from our attempt to constrain functional data by structural clusters emerging from measurements of anatomical edges and circuits between brain areas. Hence, our goal here is to establish a way to constrain factor analytic models of functional coactivation with structural properties of the brain.

The outline of this article is as follows. First, we propose a pipeline to allow the information in the structural data to guide the inference of functional coactivations. We then study the effectiveness of our pipeline by performing a set of simulation studies that target different plausible scenarios for both structural and functional connectivity profiles. Having established that we can recover different structural and functional profiles when the true state of the underlying variables is known, we turn to experimental data when such information is not available. We provide model fits to a large set of empirical data as proof of concept that our pipeline could be used effectively on real data. We also provide a preliminary analysis of structural-functional-behavioral links in an effort to assess the plausibility of our proposed pipeline and its extensions to more integrative modeling approaches.

## THE PROPOSED METHOD

**Figure 1.**
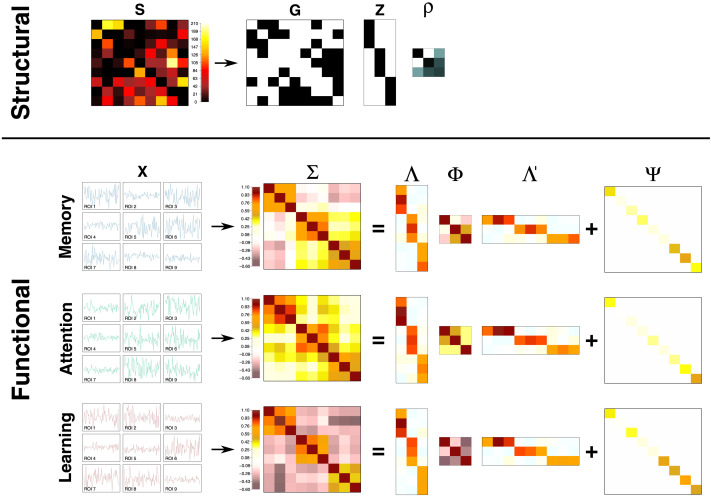
Two-step pipeline for structural and functional data analysis. An illustration with *P* = 9 ROIs and *K* = 3 clusters (3 ROIs per cluster). The first row describes the first step of the pipeline in which a nonparametric Bayesian clustering method is applied to the streamline counts matrix ***S***. This produces a cluster assignment matrix ***Z***, a cluster connectivity matrix ***ρ***, and a binary ROI-to-ROI connectivity matrix ***G***. The other rows illustrate the second step in which a factor analysis model is applied to a correlation matrix of fMRI data measured during different cognitive tasks (e.g., memory, attention, and learning tasks). This produces a factor-ROI loading matrix **Λ**, a factor correlation matrix **Φ**, and a uniqueness (the proportion of variance unexplained by factors) matrix **Ψ**. In the second step, factors are defined as the clusters from the first step and the factor structure is informed by the clustered structural network (i.e., ***Z*** informs the shape of **Λ**). Task-dependent coactivations of brain factors (clusters) are captured by differences in **Φ** matrices. See the *Models* section for a detailed description of the models in the pipeline.

In an effort to constrain functional connectivity analyses by structural data, we propose a two-step pipeline, illustrated in Figure 1. First, one estimates the structural connectivity of ROIs from measurements of structural brain data, such as streamline counts obtained with diffusion-weighted imaging (DWI) or diffusion tensor imaging (DTI). Second, this estimated structural description of the brain is used to specify the number of factors and a factor loading structure in subsequent **factor analysis** (FA). The goal of the FA is to relate the clusters identified in the first step to patterns in functional data that might be obtained in fMRI experiments. Central to our pipeline is the notion that different types of tasks might recruit different clusters. Hence, our pipeline is intended to be able to interpret the functional role of clusters identified in the structural analysis.

### Step 1: Nonparametric Clustering for Structural Connectivity

As our primary goal is to use structural data to guide the analysis of functional data, we first need to properly characterize the structural relationships that exist between a set of ROIs. However, a complication is that different ROIs may be more strongly connected with one another than with other ROIs, creating a subset of ROIs that cluster together. Additionally, these clusters may have ROIs that communicate information to other clusters, in which case these ROIs communicate within and between clusters. A final complication is that we may have no a priori knowledge about how many clusters might be present in a particular brain.

To flexibly address these issues, we will use a nonparametric Bayesian clustering method to analyze structural data, creating a map from structural connectivity data to a set of matrices that describe the latent cluster structure (as illustrated in the top panel in Figure 1 and the Nonparametric Clustering section). Our approach does not require us to specify a fixed number of clusters, and instead, we can simply let the number of clusters grow depending on what patterns occur in the data. The basic strategy of our clustering approach is to (a) identify how many clusters best describe a particular brain, (b) identify which ROIs are grouped into which clusters, and (c) estimate the strength of connections within and across clusters.

### Step 2: Factor Analytic Approach for Functional Connectivity

The next step is to relate the clusters identified in the structural analysis to patterns that might occur in functional connectivity data. To do this, we rely on FA models to characterize the covariances/correlations among ROIs with the variation of latent factors (as illustrated in the bottom panel of Figure 1 and the Exploratory and Confirmatory Factor Analysis section). In our pipeline, the latent factors themselves will be connected to the clusters identified in the structural analysis, which will allow us to determine the degree to which each cluster is recruited during different tasks. One advantage, illustrated in Figure 1, is that a cluster may have a stronger contribution to the functional data, depending on the specific task being performed. In the first two rows, a network might facilitate memory or attention performance, but in a learning task that involves both memory and attention, the functional coactivation matrices would have contributions from both tasks. By mapping the clusters into cognitive tasks in this way, and with an appropriate set of tasks, the hope is that one could isolate the individual cognitive components of each task. Factor analytic models decompose observed covariance/correlation matrices into the following three components: (a) the relationships (memberships and strengths of edges) between ROIs and factors, (b) the correlations among the various factors (i.e., clusters), and (c) the proportion of variance within the fMRI data that is unexplained by the underlying factors.

### Models

In this section, we formally define the models used within the pipeline. We first define the nonparametric clustering model, and then provide the details of the two types of FA models we will use in the analyses below.

#### Nonparametric clustering.

To first provide a clustering-based representation of the brain data, we implement a nonparametric clustering method proposed by Hinne et al. (2015) that applies to a streamline counts matrix ***S*** obtained with DWI/DTI. This method consists of the **infinite relational model** (IRM; Andersen et al., 2014; Kemp, Tenenbaum, Griffiths, Yamada, & Ueda, 2006; Mørup, Madsen, Dogonowski, Siebner, & Hansen, 2010; Xu, Tresp, Yu, Yu, & Kriegel, 2007) as a connectivity-based clustering prior and the **Dirichlet compound multinomial distribution** as the likelihood function. Assuming we have *P* ROIs, the IRM is a nonparametric Bayesian model that applies to a binary square (*P* × *P*) matrix of connectivity information ***G*** where its element in the *i*th row and *j*th column is denoted *g*_*ij*_ and takes on a value of 1 if ROIs *i* and *j* are connected, and 0 otherwise. A diagonal element *g*_*ii*_ (*i* = 1, …, *P*) represents the link from an ROI *i* to itself, and this is defined to be 0. The IRM assumes that the connectivity information within ***G*** is a result of a multiplicative interaction between a cluster membership matrix ***Z***, and a cluster connection probability matrix ***ρ***. Letting *K* denote the number of clusters, the cluster membership matrix ***Z*** is a (*P* × *K*) binary-valued matrix for which the individual elements *z*_*ik*_ equal 1 if the *i*th ROI is a member of cluster *k* and they equal 0 if ROI *i* is not included in cluster *k*. Because each ROI is assumed to be included in only one cluster, each row vector of ***Z*** has only a single element with a value of 1, and all the other elements are equal to 0. As a consequence, the column-wise sum of ***Z*** gives the number of ROIs contained within a given cluster. The cluster connectivity matrix ***ρ*** is a (*K* × *K*) symmetric matrix whose diagonal elements correspond to the strength (probability) of the within-cluster connectivity, and whose off-diagonal elements correspond to the strength of the between-cluster connectivity.

The primary motivation for using the IRM is that we do not need to specify the number of clusters a priori. Instead, the complexity of the data and the specific relationships between the ROIs in the structural data largely contribute to the number of clusters that will be extracted from the application of the IRM. Because the number of clusters will be directly extracted from the data, our goal in fitting the IRM to data will be to estimate dimensions of ***Z*** and its binary elements. To do this, we specify a distribution over all possible realizations of ***Z*** through the so-called **Chinese restaurant process** (CRP; Aldous, 1985; Gershman & Blei, 2012). To describe this process, we rely on the classic scene from a Chinese restaurant: Consider a restaurant with an unlimited number of tables and an unlimited number of seats per table. Customers enter the restaurant one by one. The first customer takes a seat at the first table, which constitutes the construction of the first cluster. The second customer is then presented with a choice: They may take a seat at the same table as the first customer, or they may decide to sit at another, unoccupied table. This process continues, with each new customer choosing to take a seat either at any of the tables already occupied by earlier customers, or at a new unoccupied table. Importantly, the number of occupied tables is not limited and continues to grow as needed. The probability of a new customer taking a seat at an occupied table is proportional to the number of customers currently sitting at the table. Namely, if there are *m*_*k*_ customers at table *k*, the probability that a new customer chooses table *k* is $\frac{m_{k}}{p-1+\xi }$, whereas the probability that the same customer chooses a new unoccupied table is $\frac{\xi }{p-1+\xi }$. The parameter *ξ* is a tuning parameter that dictates the dispersion of customers across tables, where larger values of *ξ* are associated with more tables being occupied by at least one person (i.e., more total tables). The CRP probability density function of ***Z*** is given as the following (Hinne et al., 2015; Mørup et al., 2010):

$$P\left(Z|\xi \right)=\xi^{K}\frac{\Gamma \left(\xi \right)}{\Gamma \left(\xi +K\right)}\prod_{a}\Gamma \left(n_{a}\right),$$ (1)

where *K* is the current number of tables (which can change during the model fitting procedure), *n*_*a*_ is the number of customers currently assigned to table *a*, and Γ(*x*) = (*x* − 1)! is the gamma function. Applying this analogy to our application of structural connectivity data, customers correspond to ROIs, and tables correspond to clusters.

With the CRP as a prior distribution for the cluster assignment matrix ***Z***, the IRM can be expressed as follows:

$$\begin{array}{l} Z|\xi ∼CRP\left(\xi \right),\\ \rho_{ab}|\alpha ,\beta ∼\text{Beta}\left(\alpha ,\beta \right),\\ g_{ij}|\rho ,Z∼\text{Bernoulli}\left(z_{i}\rho z_{j}^{\prime }\right), \end{array}$$ (2)

where ***z***_*i*_ is the *i*th row vector of ***Z*** and *ρ*_*ab*_ is the entry in the *a*th row and *b*th column of ***ρ***. For the cluster connectivity matrix ***ρ***, the choice of a *Beta* prior is made simply for convenience and flexibility of the Beta distribution, but any distribution over the space [0, 1] is possible. Together, the cluster membership matrix and the cluster connectivity matrix determine the probability of two ROIs being structurally connected. Notice that if ROIs are in the same cluster, they will also have the same connection probabilities. For example, if ROIs *i*, *j*, and *l* are in the same cluster *a*, the connection probability of ROIs *i* and *j* is *ρ*_*aa*_, which is equal to the connection probability of ROIs *i* and *l*. If an ROI *h* is assigned in another cluster *b*, the three ROIs in cluster *a* have the same probability of having an edge with *h*, which is *ρ*_*ab*_.

**Figure 2.**
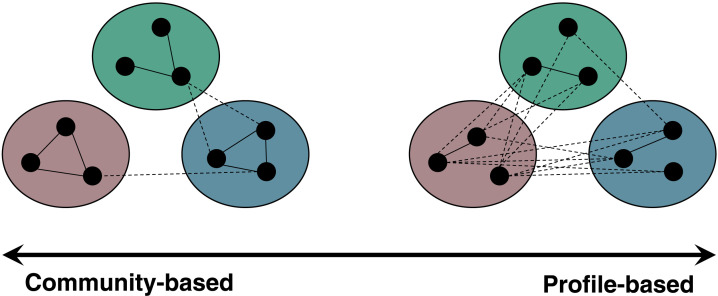
Two types of structural clusters. Adopted from Figure 2 in Hinne et al. (2015). The colored circles represent clusters, whereas the small black circles within the colored circles represent ROIs. Solid lines show within-cluster edges and dashed lines show between-cluster edges.

One important feature of the IRM as a clustering prior for structural connectivity data is that the IRM can capture two prominent types of clusters (Hinne et al., 2015): community-based and profile-based (Figure 2). In a **community-based cluster**, ROIs are densely connected within the cluster that they are a member of, but are less likely to be connected to other ROIs that are members of different clusters. The IRM creates this type of cluster when the diagonal entry of ***ρ*** has a large value but its corresponding off-diagonal entries usually have small values. For a **profile-based cluster** (also called connectivity-based clusters; Anwander et al., 2007; Cloutman & Lambon Ralph, 2012; Johansen-Berg, Behrens, Sillery, et al., 2004; Johansen-Berg, Behrens, Robson, et al., 2004; Mars et al., 2011), ROIs within the cluster are not necessarily highly interconnected but tend to be connected to other specific ROIs with similar connectivity profiles. The IRM creates a profile-based cluster by having off-diagonal entries with large values to some other clusters common to a set of ROIs (and/or diagonal entries of ***ρ*** with low values, although not necessarily). It is also worth noting that a community-based cluster can be understood as a special case of a profile-based cluster, as nodes within a community have similar connectivity profiles. Many earlier clustering methods such as the Infomap algorithm (Rosvall & Bergstrom, 2008) cannot account for profile-based clusters when applied to a streamline count data for the structural connectivity (Hinne et al., 2015). Importantly, structural clusters may have features of both community-based and profile-based clusters. Hence, clustering methods may miss a great deal of information on the underlying structure if they are not capable of capturing different types of clusters.

To connect the IRM-based clustering prior to some streamline structural connectivity matrix ***S***, Hinne et al. (2015) introduced the Dirichlet compound multinomial distribution as a likelihood function for the individual rows of ***S***, which we denote as ***s***_*i*_ for the *i*th row of ***S***. Given the structural connectivity matrix ***G***, the row of streamline data

$$s_{i}|g_{i},\delta_{0},\delta_{1}∼DM\left(\delta_{1}g_{i}+\delta_{0}\left(1_{p}-g_{i}\right)\right),$$ (3)

where *DM*(***δ***) represents the Dirichlet compound multinomial distribution with a *p*-dimensional vector ***δ*** as its parameter, ***g***_*i*_ is the *i*th row of ***G***, **1**_*p*_ is a *p*-dimensional vector of ones, and *δ*_1_ and *δ*_0_ are Dirichlet parameters for present and absent connections, respectively. The Dirichlet parameter is set to *δ*_1_ if *g*_*ij*_ = 1 and *δ*_0_ if *g*_*ij*_ = 0. When *δ*_1_ is larger than *δ*_0_, the model will generate larger streamline counts for *s*_*ij*_ with *g*_*ij*_ = 1, and smaller counts for the others. As its name implies, the model is equivalent to the combination of a multinomial distribution for ***s***_*i*_ given a *p*-dimensional probability vector ***y***_*i*_ and a Dirichlet distribution for ***y***_*i*_ given ***G***, *δ*_0_, and *δ*_1_, but with ***y***_*i*_s integrated out. Consequently, we can express Equation 3 as

$$\begin{array}{l} s_{i}|y_{i}∼\text{Multinomial}\left(y_{i}\right),\\ y_{i}|G,\delta_{0},\delta_{1}∼\text{Dirichlet}\left(\delta_{1}g_{i}+\delta_{0}\left(1_{p}-g_{i}\right)\right). \end{array}$$ (4)

Integrating the ***y***_*i*_s out, the probability density function of the Dirichlet compound multinomial distribution of ***S*** can be expressed as (Hinne et al., 2015) the following:

$$P\left(S|G,\delta_{1},\delta_{0}\right)=\prod_{i}\left[\frac{\left(\sum_{j}s_{ij}\right)!}{\prod_{j}s_{ij}!}\frac{\Gamma \left(\sum_{j}b_{ij}\right)}{\Gamma \left(\sum_{j}\left(b_{ij}+s_{ij}\right)\right)}\prod_{j}\frac{\Gamma \left(b_{ij}+s_{ij}\right)}{\Gamma \left(b_{ij}\right)}\right],$$ (5)

where *b*_*ij*_ = *δ*_1_*g*_*ij*_ + *δ*_0_(1 − *g*_*ij*_).

Equation 2 and Equation 3 define a hierarchical Bayesian model that we can use to explain the streamline count matrix ***S*** with the structural connectivity matrix ***G*** and the cluster membership matrix ***Z***. The joint posterior distribution of ***G*** and ***Z*** is

$$P\left(G,Z|S,\delta_{0},\delta_{1},\alpha ,\beta ,\xi \right)\propto P\left(S|G,\delta_{0},\delta_{1}\right)P\left(G|Z,\alpha ,\beta \right)P\left(Z|\xi \right),$$ (6)

and this gives

$$P\left(G|Z,S,\delta_{1},\delta_{0},\alpha ,\beta \right)\propto P\left(S|G,\delta_{1},\delta_{0}\right)P\left(G|Z,\alpha ,\beta \right),$$ (7)

$$P\left(Z|G,S,\alpha ,\beta ,\xi \right)\propto P\left(G|Z,\alpha ,\beta \right)P\left(Z|\xi \right).$$ (8)

In the above equations, *P*(***Z***|*ξ*) and *P*(***S***|***G***, *δ*_0_, *δ*_1_) are given in Equation 1 and Equation 5 and *P*(***G***|***Z***, *α*, *β*) can be derived as (Hinne et al., 2015; Kemp et al., 2006; Mørup et al., 2010) the following:

$$\begin{array}{l} P\left(G|Z,\alpha ,\beta \right)=\int P\left(G|\rho \right)P\left(\rho |\alpha ,\beta \right)d\rho \\ \;=\prod_{a\geq b}\frac{\text{Beta}\left(\alpha +M_{+}\left(a,b\right),\beta +M_{-}\left(a,b\right)\right)}{\text{Beta}\left(\alpha ,\beta \right)}, \end{array}$$ (9)

where *M*_+_(*a*, *b*) is the number of edges (links) between clusters *a* and *b* and *M*_−_(*a*, *b*) is the number of non-edges (non-links) between clusters *a* and *b*. The joint posterior distribution of ***G*** and ***Z*** can be approximated by iteratively updating (a) ***G*** by a Metropolis sampler applied to Equation 7 (Hinne et al., 2015; Hinne, Heskes, Beckmann, & van Gerven, 2013) and (b) ***Z*** with Equation 8 applying a Gibbs sampling method for the IRM combined with the split-merge Metropolis-Hastings updates (Hinne et al., 2015; Jain & Neal, 2004; Kemp et al., 2006; Mørup et al., 2010). The **maximum a posteriori** (MAP) estimate of the cluster connectivity matrix ***ρ*** (which is integrated out in Equation 9 and so not sampled) is (Kemp et al., 2006; Mørup et al., 2010)

$$\rho^{MAP}=\frac{M_{+}\left(a,b\right)+\alpha }{M_{+}\left(a,b\right)+M_{-}\left(a,b\right)+\alpha +\beta }.$$ (10)

With the updating scheme above, we obtain posterior estimates of ***G***, ***Z***, and ***ρ***. These matrices provide us with the information on how ROIs are mutually connected, how they construct the clustered network, and how ROIs within and between clusters are interconnected. In the second step of our pipeline, we will incorporate this information into our functional connectivity model such that the changes in the functional connectivity in the brain can be isolated when a subject performs multiple tasks.

#### Exploratory and confirmatory factor analysis.

The second step of our pipeline is to use the structural data to impose constraints on the analysis of functional connectivity data. Let ***x***_*n*_ denote a *P*-dimensional functional data vector of *P* ROIs at time point *n* and ***X*** denote the functional connectivity data matrix with ***x***_*n*_ as its *n*th row. We first assume that ***X*** has been standardized, meaning that ***X*** has a mean of 0 and has unit variance. The standard linear FA model assumes that a *K*-dimensional latent variable ***η***_*n*_ (with *P* >> *L*) generates ***x***_*n*_ as

$$x_{n}=\Lambda \eta_{n}+\epsilon_{n},$$ (11)

where *ϵ*_*n*_ is a *P*-dimensional error term. Letting 𝒩_*P*_(*a*, *b*) denote a multivariate normal distribution of dimension *p*, mean vector *a*, and correlation matrix *b*, it is further assumed that ***η***_*n*_ ∼ 𝒩_*K*_(0, **Φ**) where **Φ** is a (*K* × *K*) factor correlation matrix, *ϵ*_*n*_ ∼ 𝒩_*P*_(**0**, **Ψ**) where **Ψ** is a (*P* × *P*) diagonal matrix of error variance (also called *uniqueness*, the proportion of variance in ***x***_*n*_ unexplained by the factor structure), and ***η***_*n*_ and ***ϵ***_*n*_ are independent. This implies that the covariance matrix **Σ** (same as the correlation matrix as ***X*** is assumed to be standardized) can be decomposed as follows:

$$\Sigma =\Lambda \Phi \Lambda^{\prime }+\Psi .$$ (12)

The utility of FA models is to decompose the correlation matrix **Σ** to find a systematic latent structure underlying the data represented by **Λ**. The marginal distribution of ***x***_*n*_ is given as ***x***_*n*_ ∼ 𝒩_*p*_(**0**_*p*_, **ΛΦΛ**′ + **Ψ**). The model likelihood can be obtained from this distribution and a conventional estimation method such as the maximum likelihood estimation (MLE) can be conducted to find the optimal solution of **Λ**, **Φ**, and **Ψ** (Jöreskog, 1967, 1969; Lawley, 1943). Bayesian estimation can be conducted based on the same marginal distribution, or the conditional distribution of the model that can be expressed as ***x***_*n*_ ∼ 𝒩_*p*_(**Λ*η***_*n*_, **Ψ**) and ***η***_*n*_ ∼ 𝒩_*K*_(0, **Φ**) and prior distributions on the model parameters (Conti, Frühwirth-Schnatter, Heckman, & Piatek, 2014; Kang, Yi, & Turner, 2022; Song & Lee, 2012).

The novelty of our approach centers on using the structural data to impose structure on the latent factors, particularly on the factor loading matrix **Λ**. Here, we define the latent factors to be the same clusters obtained in Step 1 of our pipeline that identifies the structural connectivity network, and thus, the number of factors is *K* estimated from the CRP-based clustering prior. In addition, the factor loading structure itself is determined by the cluster membership matrix ***Z*** such that the estimate for *λ*_*ij*_ is expected to be large when *z*_*ij*_ = 1, but is expected to be close to 0 or even fixed to 0 when *z*_*ij*_ = 0. This constraint is imposed for all functional data across tasks, and so the model maintains the same factor loading structure for all tasks, but not necessarily the same factor loading estimates. The factor correlation matrix **Φ** shows which brain clusters (defined as factors) strongly coactivate during a cognitive task. Unlike the loading matrix, the correlations are expected to largely differ by subject and by task, capturing both individual- and across-task differences in functional connectivity. Despite their conceptual similarity, **Φ** need not to be similar to ***ρ***. Having these two matrices diverge can be advantageous when modeling both structural and functional connectivity because clusters with many intercluster edges between them may not have strong interactions during a specific cognitive task. Also, even a few physical edges between clusters (i.e., clusters that are not strongly connected in the structural network) may produce a strong functional coactivation depending on the nature of the task.

In this step of our pipeline, we suggest using both exploratory factor analysis (EFA) and confirmatory factor analysis (CFA) models. The EFA approach imposes a weaker constraint on **Λ** in the sense that cross loadings (*λ*_*ij*_ corresponding to *z*_*ij*_ = 0) are allowed to be nonzero. The EFA models achieve the ideal factor structure (e.g., in our case, the clustered structure provided by the first step of our pipeline) by factor rotation. Note that, in FA, there are infinitely many solutions for **Λ** and **Φ** that are mathematically equivalent. To see this, consider a (*K* × *K*) rotation matrix ***T*** that satisfies ***TT***′ = ***I***_*K*_ where ***I***_*K*_ is a (*K* × *K*) identity matrix. Then, we have the following equivalence for Equation 12:

$$\begin{array}{l} \Sigma =\Lambda \Phi \Lambda^{\prime }+\Psi \\ \;=\Lambda TT^{\prime }\Phi TT^{\prime }\Lambda^{\prime }+\Psi \\ \;=\Lambda^{*}\Phi^{*}{\left(\Lambda^{*}\right)}^{\prime }+\Psi , \end{array}$$ (13)

where Λ* = **Λ*T*** and Φ* = ***T***′**Φ*T***. That is, Λ* and Φ* construct another FA solution for **Σ**. This identifiability issue is known as *rotational invariance*, but is solved in EFA by finding the rotation matrix ***T*** that leads us to a simple and interpretable factor loading structure.

There are different rotation methods for different definitions of what “simple structure” means, but in our pipeline, we implement target rotation (Browne, 1972a, 1972b; Zhang, Hattori, Trichtinger, & Wang, 2019). In general, target rotation produces the factor loading solution that is closest to the input target matrix ***B***. The target matrix has zero and nonzero elements and is of the same size as the factor loading matrix. For example, in the case assumed in Figure 1 in which we have three factors (i.e., clusters in our pipeline) measured by different three ROIs (nine ROIs in total), the following target matrix ***B*** can be used to achieve the assumed underlying structure:

$$B^{\prime }={\left[\begin{array}{ccccccccc} 1 & 1 & 1 & 0 & 0 & 0 & 0 & 0 & 0\\ 0 & 0 & 0 & 1 & 1 & 1 & 0 & 0 & 0\\ 0 & 0 & 0 & 0 & 0 & 0 & 1 & 1 & 1 \end{array}\right]}^{\prime }.$$ (14)

The corresponding target rotation matrix ***T*** can be found by minimizing the objective function (Browne, 2001) given as *f*(**Λ**) = $\sum_{j=1}^{k=1}$ ∑_*i*∈*I*_*j*__(*λ*_*ij*_ − *b*_*ij*_)^2^ where **Λ** = **Λ**_0_***T*** is the rotated solution obtained from the initial factor loading solution **Λ**_0_, *λ*_*ij*_ and *b*_*ij*_ are the (*i*, *j*) entries of **Λ** and ***B***, respectively, and *I*_*j*_ is the set of row indices of specified target loadings *b*_*ij*_ in column *j* (in this case, *I*_*j*_ = {1, ⋯, 9} for all *j*). Thus, the target rotation attempts to make the structure of **Λ** closer to the specified loading structure in ***B***. This makes the loadings in **Λ** corresponding to 0 in the target matrix as close to 0 as possible. However, this is not a strong constraint and the EFA can produce a large loading *λ*_*ij*_ even if *b*_*ij*_ = 0 when data imply a large association between an ROI and a factor. To reach the functional factor structure induced by the obtained structural network, we use the cluster assignment matrix ***Z*** as the target matrix. Hence, the structural data literally guide the analysis of our functional data.

In contrast to the EFA model, the CFA model does not use factor rotation but imposes an even stronger constraint on **Λ**, such that all cross loadings are fixed to 0. Therefore, the factor structure in the CFA approach is fully determined by ***Z***. Because nonzero cross loadings are not allowed, covariations between ROIs in different clusters should be captured solely by off-diagonal elements of **Φ**. In practice, this might be too restricted to account for functional connections between ROIs, particularly when there are large between-cluster connections or when noise in functional activation is large.

#### Choosing between EFA and CFA.

The choice of using EFA or CFA may depend on a few different criteria. One theoretical criterion is whether we can assume that cluster memberships of ROIs in functional data are exactly the same as those in structural data. This criterion would imply that the structural connectivity network should strongly constrain the functional brain activation. If so, there is no need to consider adding additional edges (that are not present in the structural network) to the functional connectivity network, such as an edge from an ROI to the other clusters in which the ROI is not included. In this case, there is no need to allow nonzero cross loadings and the CFA approach is preferred. Here, the CFA would explain all the intercluster functional coactivations through the factor correlations that represent indirect functional coactivations of ROIs through clusters. By contrast, if this theoretical constraint is too strong, then EFA is preferred so that ROIs can potentially have direct edges (i.e., nonzero cross loadings) to the other clusters in which it is not included in the (estimated) structural network. In this case, the functional coactivations of clusters can be accounted for by combinations of cross loadings and factor correlations. This variation can be practical because (a) measurements of the structural network based on neuroimaging might be too focused on a specific type of connection such as cortico-cortical connections, and (b) models for structural connectivity may miss some ROI-to-ROI edges because of error and uncertainty in estimation. Thus, the estimated structural network, which is used to constrain functional data, could be too restrictive to effectively account for functional coactivations.

One practical criterion is whether the FA models can be properly estimated. The estimation algorithm can sometimes produce improper solutions such as nonconvergence, negative variances, nonpositive-definite covariance/correlation matrix predicted from a model fit (Chen, Bollen, Paxton, Curran, & Kirby, 2001; Heywood, 1931). For example, if a factor structure specified by an estimated structural network is too restrictive, a CFA model may not be able to adequately account for functional coactivations because it has to do this only with a factor correlation matrix. Also, if an estimated structural network has a severe discrepancy with the underlying correlation structure of functional data, then the factor structure has severe misspecification and potentially produces Heywood cases (i.e., some variance estimates are 0 or negative). Heywood cases can also occur because of the underidentification of a model or because of outliers.

## SIMULATION STUDY: PARAMETER RECOVERY

In this section, we aim to validate the models contained within our pipeline to provide some assurances that we can accurately recover specific structural and functional patterns when they exist. Although parameter recovery of the IRM was presented in Kemp et al. (2006), the study focused on the recovery of the number of clusters when clusters have an equal number of entities and did not provide the recovery results of cluster memberships and cluster connection probabilities. Furthermore, the extension of the IRM developed by Hinne et al. (2015) for streamline count data has yet to be thoroughly examined. Here, we aim to investigate whether the nonparametric clustering approach can be accurately recovered, with a comprehensive focus on all relevant aspects of the IRM: the number of clusters, cluster memberships, and the cluster connectivity matrix.

In addition to the structural model, we also wished to verify that EFA and CFA models could be reliably recovered. If the above recovery study for the structural model were successful, then the recovery of both FA models would be independent of the clustering results because of the two-step approach used in the pipeline. As such, we divide the simulation study here into two parts; first, we examine the recovery of the structural connectivity IRM under some different plausible scenarios, and second, we examine the recovery of the EFA and CFA models under different assumptions about the factor loading structure.

### Recovery of Structural Model: IRM

#### Generating structural data.

To test the general capabilities of recovering the IRM, we tested a few different underling structural networks when constructing the synthetic data. To examine recovery over a comprehensive, but still manageable, number of conditions, we generated five sets of streamline count data with different data-generating parameters. We named the five conditions the *Data-informed*, *Independent*, *Random* (two variations), and *Single* conditions. These five conditions were intended to cover both intermediate and empirically plausible cases, as well as some extreme cases with respect to the degree of interconnections between ROIs. In the data-informed condition, we tried to generate data with an empirically plausible brain structure that was motivated by the streamline data analysis result of Hinne et al. (2015). We assumed *P* = 160 ROIs and *K* = 14 clusters similarly to Hinne et al.’s result and also determined cluster assignments and cluster connectivity values based on the estimates reported in their study. The number of ROIs in each cluster was 3, 4, 6, 7, 7, 9, 10, 10, 12, 13, 15, 19, 21, and 24. In one extreme condition—the independent condition—ROIs were constructed with the same clustered structure as in the data-informed condition, but clusters were assumed to be independent, meaning that there was no intercluster connection and all clusters were of the community-based type. In this case, the cluster connectivity matrix ***ρ*** was diagonal. The number of ROIs, the number of clusters, and the cluster assignments were the same as those in the data-informed condition.

We also examined two other extreme conditions where ***ρ*** was randomly constructed. In one random condition, the connectivity values were sampled from a *Beta*(0.1, 0.1) distribution, producing values close to either 0 or 1. This resulted in very strong or very weak within/intercluster connectivity values. In the other random condition, the elements of ***ρ*** were sampled from a *Beta*(1, 1) distribution, meaning that the cluster connectivity values were uniformly distributed between 0 and 1.

In the final extreme condition—the single condition—we assumed that all ROIs were interconnected and so they constructed a single unified cluster. In this case, ***G*** was a (*P* × *P*) matrix with all its elements being equal to 1 (except for the diagonal elements, which are defined to be 0), ***Z*** was a *P*-dimensional column vector of 1s (**1**_*p*_), and ***ρ*** was a (1 × 1) matrix with 1 as its unique element, indicating perfect within-cluster connection.

For each of the five conditions, we generated the corresponding streamline count matrix ***S*** using Equation 2 and Equation 3 with 5,000 streamlines per row for multinomial sampling and *ξ* = log(*P*), *α* = *β* = 1, *δ*_1_ = 1, and *δ*_0_ = 0.1 for tuning- and hyperparameters as used for an empirical data analysis in Hinne et al. (2015). The choice for *α* and *β* puts no information on the distribution of cluster connection probabilities (i.e., uniform distribution over [0, 1]). Also, Hinne et al. stated that the clustering method was very robust for different choices of *ξ*, and the choice of *δ*_1_ and *δ*_0_ was validated from an empirical study by Hinne et al. (2013).

#### Recovery of different structural networks.

After generating synthetic structural data, we fit the nonparametric Bayesian clustering model to the structural data. We implemented a Gibbs sampling procedure (Hinne et al., 2015; Kemp et al., 2006; Mørup et al., 2010), combined with the split-merge algorithm (Jain & Neal, 2004) to improve the efficiency of estimating the the optimal number of clusters. We ran the estimation algorithm for 6,000 iterations with five chains and discarded the first 3,000 samples for burn-in. With the posterior samples of the clustering result in hand, we calculated the MAP estimates of ***G***. Because the number of columns in ***Z*** can vary over iteration, we recorded ***M*** = ***ZZ***′ as done in Hinne et al. (2015) and obtained its MAP estimate. Then, the cluster assignment matrix ***Z*** can be recovered from this matrix. There are different methods for post hoc decomposing of ***M***. In the method we used, we start from the first ROI (i.e., ROI 1), find all other ROIs connected to ROI 1 and assign them to cluster 1. For ROI 2, if it has already been assigned to a cluster (i.e., from the previous calculation), we move on to the next ROI, and if not, we find other ROIs connected to ROI 2 and assign them to cluster 2 in a similar way. Repeating this procedure produces the cluster assignment information as the ***Z*** matrix. In this method, we consider ROIs *i* and *j* connected if $\hat{m}$_*ij*_ = 1, where $\hat{m}$_*ij*_ is the (*i*, *j*) entry of the MAP estimate of ***M***. Alternatively, ROIs *i* and *j* can be considered connected if the posterior probability of *m*_*ij*_ = 1 is greater than a cutoff value, where *m*_*ij*_ is a posterior sample of the (*i*, *j*) entry of the ***M*** matrix. A cutoff value of 0.5 gives the same result as the method we used. Finally, the MAP estimate of ***ρ*** can be obtained by Equation 10 (Kemp et al., 2006; Mørup et al., 2010).

**Figure 3.**
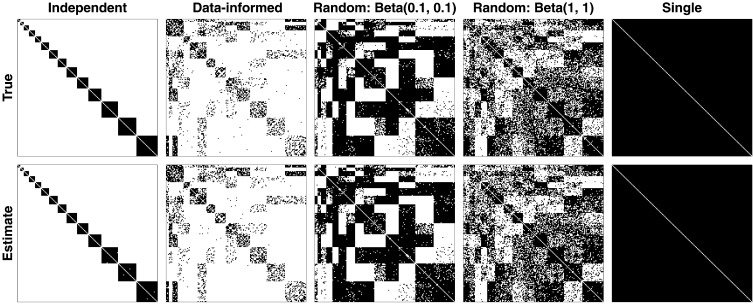
Recovery of the connectivity matrix ***G***. The top row shows the true, data-generating structural connectivity profile, whereas the bottom row shows the estimated recovery profile. We selected five plausible scenarios ranging from perfectly independent clusters (first column), representations of actual data (second column), completely randomly generated (third and fourth columns), and a single cluster (fifth column).

In Figure 3, the top row shows the true data-generating structure of the binary ROI connectivity matrices ***G*** for all five conditions, and the bottom row shows the corresponding estimates for ***G***. In each panel, rows and columns correspond to *P* = 160 ROIs and edges between ROIs are colored black. Across all panels, the estimate ***G*** matrix closely aligns with the true data-generating ***G*** in all cases.

Table 1 provides a classification result based on whether an edge was correctly or incorrectly identified. We present this result in terms of the hit rate (Hit, percentage of edges correctly recovered), miss rate (Miss = 1 − Hit), correct rejection rate (CR, percentage of non-edges correctly recovered), and false alarm rate (FA = 1 − CR) for each of the five simulated data structures (columns). Taken altogether, Figure 3 and Table 1 show that the nonparametric Bayesian clustering method can recover the ROI connectivity matrices over various connectivity structures. For all conditions except for the single condition, the number of clusters (*K* = 14) was accurately recovered. Although not shown here (implicitly shown in Figure 3), the cluster assignments ***Z*** were also correctly recovered.

**Table 1.** Recovery of the ROI connectivity matrix ***G***.

**Condition**	**Independent**	**Empirical**	**Beta(0.1, 0.1)**	**Beta(1, 1)**	**Single**
**Hit**	0.997	0.935	0.983	0.967	1.000
**Miss**	0.003	0.065	0.017	0.033	0.000
**CR**	1.000	0.986	0.984	0.941	–
**FA**	0.000	0.014	0.016	0.059	–

**Hit**: Hit rate, percentage of edges correctly recovered.

**Miss**: Miss rate, percentage of edges not recovered (i.e., estimated as non-edges).

**CR**: Correct rejection rate, percentage of non-edges correctly recovered.

**FA**: False alarm rate, percentage of non-edges not recovered (i.e., estimated as edges).

**Figure 4.**
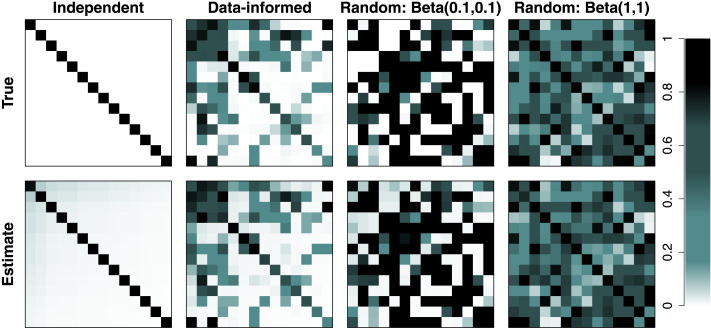
Recovery of the cluster connectivity matrix ***ρ***. The top row shows the true, data-generating connectivity profile, whereas the bottom shows the resulting estimate. We selected five plausible scenarios ranging from perfectly independent clusters (first column), representations of actual data (second column), and completely randomly generated (third and fourth columns). The scenario with a single cluster is not shown because *ρ* is not defined when there is only one cluster.

Figure 4 shows the recovery of the cluster connectivity matrix ***ρ*** for four conditions (***ρ*** is not defined in the single condition). In each panel, rows and columns correspond to *K* = 14 clusters and the within- and intercluster connectivity values are color-coded according to the legend on the far right-hand side. The Pearson correlation coefficients between the true connectivity values and their estimates were 0.998, 0.991, 0.997, and 0.980 for the independent, empirical, random with *Beta*(0.1, 0.1), and random with *Beta*(1, 1) conditions, respectively, and there was no noticeable bias in the estimates.

### Recovery of Functional Models: EFA and CFA

Having verified that we could properly recover different types of structural connectivity profiles we examined, we could then turn to the problem of recovery for functional connectivity data.

#### Generating functional data.

For this simulation, we generated multiple functional connectivity datasets with different correlation structures. The cluster membership matrix ***Z*** that generated the structural data also determined the factor loading structure (the distribution of zero and nonzero loadings) in the sense that *λ*_*ij*_ is nonzero if *z*_*ij*_ ≠ 0 and it is zero if *z*_*ij*_ = 0. The nonzero factor loadings were sampled from a normal distribution with its mean and standard deviation proportional to the within-cluster connectivity values in ***ρ***. For example, if the within-cluster connectivity for cluster *a* is *ρ*_*aa*_ = 0.5 and ROI *i* is in this cluster, we sampled *λ*_*ij*_ from a normal distribution with mean of 0.5 and standard deviation of 0.05 (= *ρ*_*aa*_/10). Thus, if cluster *a* has a strong (weak) within-cluster connectivity (i.e., *ρ*_*aa*_ is close to 1), ROIs within this cluster have large (small) factor loadings to factor *a*, which implies that the factor *a* explains large (small) proportions of variances in functional activation of ROIs assigned to the corresponding cluster. Because we assumed that the structural connectivity network is one of the physical bases of the functional connectivity network, the same clustered structure in **Λ** remains the same for all functional connectivity conditions.

As such, to generate different plausible conditions, our focus was on the **Φ** matrix. We generated five different **Φ** matrices, defining five simulation conditions: *Independent*, *Random*, and three Practically motivated (*Practical*) conditions. In the independent condition, we generated data by assuming that the factors were fully independent and had no factor correlations. In the random condition, we randomly selected the factor correlation values from a truncated normal distribution with zero mean, standard deviation of 0.25, and range of [−0.6, 0.6]. The choice of the generating distribution was to produce a fully random but still positive-definite correlation matrix. For the other three (practical) correlation conditions, we generated **Φ** matrices based on ***ρ*** in the data-informed condition in the structural connectivity simulation above. In our data-generating ***ρ***, there are both large and small (noise-level) connectivity values, and the three conditions differ on the treatment of those large and small values within ***ρ***.

In the practical 1 condition, the factor correlation matrix **Φ** was defined directly by transforming ***ρ*** so that the factor correlation values were proportional to intercluster connectivity values. To obtain **Φ**, we first transform ***ρ*** by the formula Φ* = ***D***^−1^***ρD***^−1^, where ***D*** is a diagonal matrix with its entries being square roots of the diagonal entries of ***ρ***. When applied to a covariance matrix, this transformation results in the corresponding correlation matrix. However, because ***ρ*** is not a correlation matrix and its diagonal entries can take values between 0 and 1 (as within-cluster connectivity values), Φ* is not a correlation matrix (its off-diagonal entries can be larger than 1) and also it is not a positive-definite matrix. To define a correlation matrix from Φ*, we added small random values sampled from *N*(0.005, 0.05) to the diagonal entries of Φ* and transform the matrix by the covariance-to-correlation formula. Note that here we allowed negative random values for this procedure to avoid weakening the structure of ***ρ*** too much. If only positive random values were used (e.g., those from *U*(0, 0.05)), the procedure tends to produce smaller correlation values in the outcome matrix. This procedure was repeated until the resulting matrix has positive eigenvalues, and the outcome of this procedure was used as **Φ** for the practical 1 condition. This procedure led us to a correlation matrix **Φ** that has a similar structure as ***ρ***; the Pearson correlation between lower triangular entries of ***ρ*** and those of the resulting **Φ** was 0.939.

In the practical 2 condition, we aimed to examine the case in which functional coactivations would be inversely proportional to structural connections; few edges (but not too few) in the structural network can produce high functional connectivity, and/or many edges in the structural network can produce low functional connectivity. First, we considered *ρ*_*ab*_ large if its value was greater than 0.3 and otherwise *ρ*_*ab*_ was considered small (too few, or noise-level). As a result, all diagonal entries of ***ρ*** were considered large (as its minimum value was about 0.315 in the data-informed condition in our simulation study) and about 20% of the off-diagonal entries (between-cluster connectivity) values were considered large. The factor correlation matrix for this condition was generated as follows. First, if *ρ*_*ab*_ was large, the corresponding element in **Φ** (denoted by *ϕ*_*ab*_) was sampled from a normal distribution where the mean of the distribution is set to 1 − *ρ*_*ab*_ and the standard deviation is *ρ*_*ab*_/10. If *ρ*_*ab*_ was small, we considered the cluster connections were due to noise, and accordingly, we assign a small random value sampled from *N*(0, 0.05) to *ϕ*_*ab*_.

Lastly, in the practical 3 condition, we examined an even more extreme version of the practical 2 condition in which small noise-level structural connections produce large functional coactivations while ROIs with a large number of structural edges have only noise-level coactivations. This structure does not have a reasonable interpretation, just as the randomly generated factor correlation matrix, but was included in our simulation for an exhaustive examination of the recovery. In this condition, *ϕ*_*ab*_ corresponding to a noise-level *ρ*_*ab*_ was given a relatively large value sampled from a truncated normal distribution with mean of 0.3, standard deviation of 0.3, and range of [0.1, 0.6], and *ϕ*_*ab*_ corresponding to a large *ρ*_*ab*_ was given a small random value sampled from *N*(0, 0.05).

Given **Λ** and **Φ** for each condition, we generated a uniqueness *ψ*_*ii*_ for ROI *i* (*i* = 1, …, *P*) by 1 − *diag*(**ΛΦΛ**′)_*i*_ where *diag*(***A***)_*i*_ denotes the *i*th diagonal element of a square matrix ***A***. This led us to have **Σ**, produced by Equation 12, with unit diagonal elements. Lastly, for each of the five conditions, we generated a functional data matrix ***X*** with *N* = 2*P* = 320 time points (twice the number of ROIs *P*) from a multivariate normal distribution with zero mean vector and **Σ** as a correlation matrix.

We generated these functional datasets (for the five functional connectivity conditions) for each of the structural connectivity conditions examined above, but except for the single condition because no clustered structure was defined for this condition. This produced 5 × 4 = 20 combinations of structural and functional connectivity conditions. However, as the nonparametric Bayesian clustering method was able to accurately recover the cluster assignments (the number of clusters and which ROIs are included in each of the clusters), recovery for functional connectivity data can be carried out independently of the structural connectivity conditions.

#### Recovery of different functional networks.

The structure we estimated for ***Z*** in the data-informed condition above was used to constrain the solution for the factor loading matrix **Λ** in our second step to analyze the functional data. We examined both EFA and CFA models with the number of factors specified as the obtained number of clusters. For the EFA model, we used the estimate of ***Z*** as a target matrix for target rotation. For the CFA model, we constrained **Λ** by ***Z*** and fixed cross loadings to 0. We fitted the models with the MLE method and this produced the estimates of **Λ**, **Φ**, and **Ψ**.

**Figure 5.**
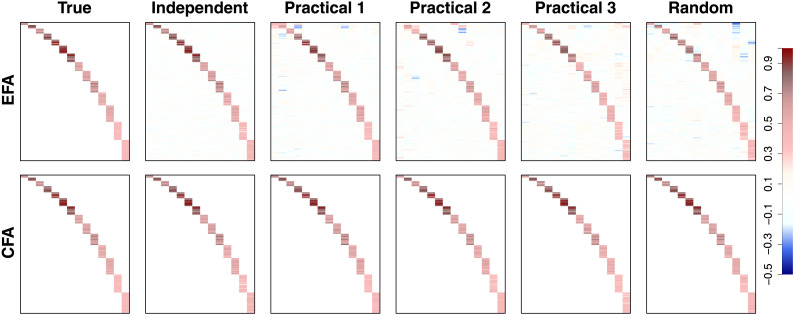
Tile plots for the recovery of the factor loading matrix **Λ**. The top row shows the recovered factor loading matrix using exploratory factor analysis (EFA), whereas the bottom shows the results using confirmatory factor analysis (CFA). The columns correspond to the various conditions in our simulation study, where the first column shows the true, data-generating factor loading matrix.

**Figure 6.**
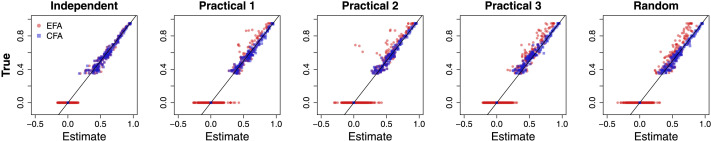
Scatterplots for the recovery of the factor loading matrix **Λ**. The estimated factor loading matrix value is shown against the true value used to generate the data for both exploratory factor analysis (EFA; red circles) and confirmatory factor analysis (CFA; blue squares) for each of the five conditions.

Figure 5 shows the recovery result of the factor loading matrix **Λ**. The leftmost panels show the true loading matrix (same for both EFA and CFA), whereas other columns show the estimated loadings for the five conditions considered. The top panels show the results from EFA, whereas the bottom panels show the results from CFA. All the true and estimated factor loading matrices are color-coded according to the legend on the far right-hand side. The true loading matrix was the same for all five conditions because we assumed that this was induced by the underlying structural connectivity network. Also, because the nonparametric Bayesian clustering method for structural connectivity analysis was able to correctly recover the number of clusters and the cluster memberships, the EFA and CFA models were fit to the functional data with the same target rotation matrix (EFA) and the same constraints on the factor loading structure (CFA). Figure 6 shows the same recovery result for the factor loading matrix, but as scatterplots. In each of the five panels, the estimated loadings were plotted on the x-axis against their true values on the y-axis. The red circles and the blue squares show the estimation results from the EFA approach and the CFA approach, respectively.

Figures 5 and 6 confirm that both EFA and CFA approaches can recover the true factor loading structures and loading values well. In CFA, all cross loadings were fixed to 0 (true values) and the other loadings with nonzero true values were recovered well without a noticeable bias. In EFA, loadings with nonzero true values were generally well recovered, but some of those loadings were underestimated (which was due to the nonzero cross loadings allowed in EFA, as explained later). Although target rotation was able to successfully recover the structure, there were some cross loadings that had large estimates. These were shown by the pale brown and blue colors in Figure 5 and the red circles horizontally distributed at *y* = 0 in Figure 6. Importantly, these nonzero cross loadings accounted for some portion of covariances in functional data that had to be captured by the factor correlation values and the loadings with nonzero true values. This explains the (underestimation) bias of the loadings in EFA mentioned above.

**Figure 7.**
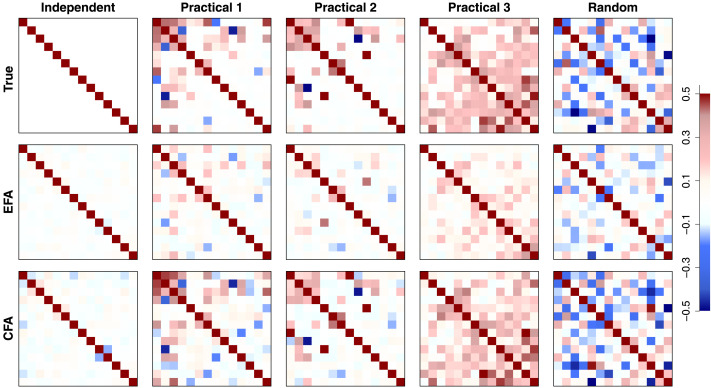
Tile plots for the recovery of the factor correlation matrix **Φ**. The first row show the true, data-generating matrix, whereas the second and third rows show the estimates obtained using either exploratory factor analysis (EFA) or confirmatory factor analysis (CFA), respectively. Each of the columns corresponds to a particular condition used in the simulation study. For visual clarity, the cells in each matrix have been thresholded at 0.5 and color-coded according to the key on the right-hand side.

**Figure 8.**
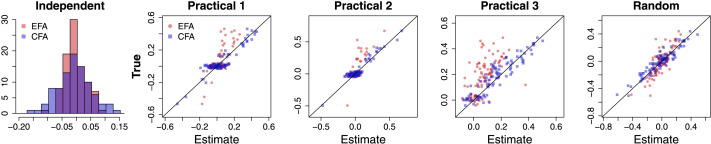
Scatterplots for the recovery of the factor correlation matrix **Φ**. Each panel shows the estimated factor correlation matrix against the true data-generating value for each of the five conditions. In the Independent condition (first column), the estimates are shown as histograms because all of the true values were set to 0. In each panel, red corresponds to the estimate obtained using exploratory factor analysis (EFA) and blue corresponds to the estimate obtained using confirmatory factor analysis (CFA).

Figure 7 shows the recovery result of the factor correlation matrix **Φ**. The top row shows the true factor correlation matrices for the five conditions. The middle and bottom panels show the recovery results from EFA and CFA, respectively. All the factor correlation matrices were color-coded according to the legend on the far right-hand side. Because most of the off-diagonal entries were lower than 0.5 while the diagonal entries were (fixed to) 1, all values higher than 0.5 were colored red for visual clarity. Figure 8 also shows the recovery result of **Φ** but as a histogram and scatterplots of off-diagonal entries. In the independent condition (the leftmost panel), true values for all the off-diagonal entries are 0 and thus the recovery result is shown by a histogram. The EFA and CFA results are color-coded by red and blue, respectively. For the other conditions, the recovery results are shown by scatterplots in which estimated correlation values are plotted on the x-axis against their true values on the y-axis. In each scatterplot, red circles and blue squares show the recovery results of the EFA and CFA approaches, respectively.

The results show that the CFA model can recover the true correlation structure and values well. For the first three conditions (Independent, Practical 1 and 2), there were many zeros in the true correlation matrices and CFA estimates were correspondingly small. The CFA model also performed well in the other conditions without a notable bias. By contrast, the EFA model tended to underestimate the correlation values. This produced a better recovery result for the independent condition because the true correlation values were 0. However, the model caused a quite large shrinkage effect in the other conditions (this will be investigated more below, with the factor loadings and uniqueness estimation results). Although the strengths of the correlation values were weaker, the EFA model was able to maintain the underlying structures and patterns of the factor correlation matrix. Thus, the factor correlation estimates from EFA models can be considered a lower bound of the functional connectivity between clusters. Taken together, this implies that we can study changes in the functional connectivity structure across tasks with both EFA and CFA approaches, and the CFA model can produce more accurate factor correlation estimates when the assumed loading structure (informed by the obtained structural connectivity) is correct.

**Figure 9.**
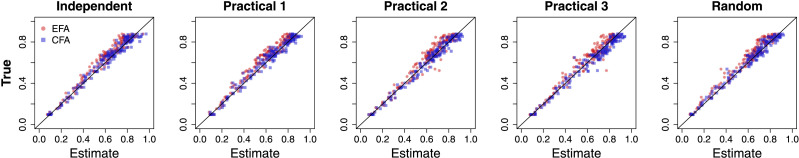
Scatterplots for the recovery of the uniqueness matrix **Ψ** (diagonal entries). Each panel shows a scatterplot of the estimates obtained using exploratory factor analysis (EFA; red) or confirmatory factor analysis (CFA; blue) for each of the five conditions examined in the simulation study. In each panel, estimates are plotted on the x-axis, whereas the true data-generating values are shown on the y-axis.

Lastly, Figure 9 shows scatterplots of the recovery result of the uniqueness matrix **Ψ**. In each of the five scatterplots, the estimated uniqueness values are plotted on the x-axis against their true values on the y-axis, where the red circles and blue squares show the results from the EFA and CFA models, respectively. Generally, both approaches recovered the uniqueness values reasonably well across the five conditions examined.

In addition to the accuracy of the estimates, one should also examine the general fits of the FA model to the synthetic data. General model fits can be examined by consistency between a sample correlation matrix ***R*** and an implied (i.e., reproduced) correlation matrix $\hat{\Sigma }$ obtained from the estimated parameter matrices: $\hat{\Sigma }$ = $\hat{\Lambda }\hat{\Phi }\hat{\Lambda }$ + $\hat{\Psi }$ (Equation 12). We computed the **standardized root mean squared residuals** (SRMR; Jöreskog & Sörbom, 1981) and the **root mean squared error of approximation** (RMSEA; Steiger & Lind, 1980) with $\hat{\Sigma }$ and ***R***. Across the five conditions, SRMR varied in the range of [0.030, 0.032] in the EFA results and in the range of [0.048, 0.051] in the CFA results. Also, RMSEA varied in the range of [0.028, 0.030] in the EFA results and in the range of [0.026, 0.028] in the CFA results. These values are lower than the upper bound for a good fit (0.08 for SRMR and 0.06 for RMSEA; Hu & Bentler, 1998, 1999), showing that the models produced good fits to the synthetic data. Because the EFA model recovered the uniquenesses well and its absolute fits to the five simulated datasets were not worse than the CFA model (they were better in EFA because of more flexibility in the loading matrix), we can conclude that the underestimation of factor correlations was due to the nonzero cross loadings estimated in the EFA result, not due to worse model fits. This can be seen from the relationship of the model parameters stated in Equation 13: Given a good model fit and well-recovered uniquenesses, misfits in the loading matrix (nonzero cross loadings) propagate to the factor correlation matrix, producing a shrinkage effect. However, the EFA model was able to maintain the factor correlation structure (with weaker correlation values), and thus, we can employ this model to study changes in a functional network across different cognitive tasks.

## EMPIRICAL APPLICATION

In this section, we apply our pipeline to empirical data and present this application in four sections. First, we provide materials and methods related to data we examine. Second, we describe the clustered structural network we obtained from applying the nonparametric Bayesian clustering method. Third, we use the obtained structural network result to analyze the functional data. Last, we also present a preliminary analysis to find a potential link from functional connectivity to behavioral measures (such as accuracy in decision-making tasks).

### Data

#### Data acquisition.

For our empirical example, we relied on the data presented in Gaut et al. (2019). However, there are some slight differences in the preprocessing pipeline in our study. In addition, we will make use of the DTI data for structural connectivity analysis, while Gaut et al. did not. Here, we summarize the data acquisition and refer the reader to Gaut et al. (2019) for additional details.

Magnetic resonance imaging (MRI) was performed using 12-channel head coil Siemens 3T Trio MRI System at the Ohio State University’s Center for Cognitive and Behavioral Brain Imagining. BOLD activity during tasks was measured using T2*-weighted echo-planar image sequence (TR = 2,000 ms, TE = 28 ms, flip angle = 72 degrees, field of view = 222 × 222, 3 × 3 × 3 mm^3^, 38 slices). The resting-state data acquisition had slightly different imaging parameters (TR = 2,500 ms, flip angle = 75 degrees, 2.5 × 2.5 × 2.5 mm^3^ resolution, 44 slices). Subjects’ structural images were acquired using T1-weighted images at a 1 × 1 × 1 mm^3^ resolution with the following imaging acquisition parameters: TR = 1,950 ms, TE = 4.44 ms, flip angle = 12 degrees, matrix size = 256 × 224, 176 sagittal slices, and 7.5-min scan time. DTI was obtained with the following imaging parameter: voxel size = 2 × 2 × 2 mm^3^, TR = 8,300 ms, TE = 85 ms, 65 slices, and 30 directions at b = 700 s/mm^2^, scan time = 4.7 min.

Each subject was asked to perform eight cognitive tasks during the 1.5-hr MRI session. Recording also included the resting-state scans, resulting in nine conditions (eight tasks and the resting state) for functional data. Runtime and description of the tasks and the resting-state recording can be found in Table 2. We have *I* = 203 subjects in total, 19 of which repeated the experiment after on average 2.8 years (standard deviation = 0.4).

**Table 2.** Description of cognitive tasks and resting-state recording. Source: Gaut et al. (2019).

**Task**	**Runtime (s)**	**Description**
**Resting state**	360	Subjects are asked to close eyes, relax, but stay awake and let their minds wonder.
**Emotional pictures (Affect)**	360	Photographs are presented, one at a time, slightly to the left or right of the center of the screen. Subjects are asked to indicate whether the picture is shifted to the left or right relative to a green dot at the center of the screen.
**Emotional faces (Empathy)**	360	Male and female faces are presented one at a time and subjects are asked to decide whether the faces are male or female. There are four task conditions based on emotions shown on the faces: neutral, happy, sad, and fearful faces.
**Episodic memory encoding (Encoding)**	304	Name and face pairings are presented on a screen and subjects are asked to determine whether the name and the face match well with each other on a 1–4 (poor to well) scale. Four face conditions are defined and used depending on whether the face is young or old and whether it is novel or has been repeated during the experiment.
**Episodic memory retrieval (Retrieval)**	252	The task is to remember the pairs of name and face used in the episodic memory encoding task and to indicate whether the face/name pair is from the previous task, new, or if the face is repeated but was paired with a different name.
**Go/No-go**	360	Images of single letters are presented and subjects are asked to push a button when the letter is one of A, B, C, D, E and not to push the button when the letter is one of X, Y, Z.
**Monetary incentive delay (Reward)**	456	The task is to press a button as quickly as possible when a cue (a white square) is presented on the screen. Subject wins/loses money depending on when and how fast they respond.
**Working memory**	354	A sequence of letters is presented. In the control task, a subject is asked to indicate whether the current letter is underlined. In the two-back memory task, a subject is asked to determine whether the current letter is the same as the one that was presented two letters ago.
**Theory of mind (ToM)**	376	Short stories and true/false statements about the stories are presented. Subjects are asked to indicate whether the given statement is true or false.

#### Data preprocessing.

The fMRI data were preprocessed using parameters (when possible) from the minimal preprocessing pipelines of the Human Connectome Project (HCP; Glasser et al., 2013). Functional brain images were realigned to compensate for head motion, spatially smoothed (2-mm FWHM Gaussian kernel), normalized using global mean, and masked with a final brain mask. Functional images were coregisterred to T-1 weighted images, normalized to the standard brain, and refined using nonlinear registration in FSL.

Functional data were denoised following the procedure outlined in Burgess et al. (2016). Data were first denoised using a high-pass filter (2,000-s cutoff). Further denoising included regression of 12 motion parameters, in addition to independent component analysis (ICA)-based denoising. Next, an additional high-pass filter with a 200-s cutoff was applied. Images were parcellated into 333 ROIs using the Gordon parcellation (Gordon et al., 2016), but we used a brain mask that overlaps these 333 ROIs, which resulted in 305 ROIs (see the Supporting Information for the list of ROIs, which was produced based on the supplementary material provided by Gordon et al., 2016).

DTI data were processed using FSL FDT. The pipeline corrects for eddy currents and estimates diffusion parameters. White matter connectivity was obtained using FDT Probtrackx 2.0 (Behrens, Berg, Jbabdi, Rushworth, & Woolrich, 2007) using the same 305 ROIs as for the functional data. FDT pipeline was run using the default parameters; each seed voxel had 5,000 streamlines drawn with a maximum length of 2,000 steps, and streamlines with sharp angle greater than 80 degrees were discarded.

### Structural Data Analysis

Our target data have *P* = 305 ROIs and *K* = 203 subjects, which is considerably larger than the data studied in the earlier empirical result from Hinne et al. (2015). Because of the computational cost of the nonparametric clustering model, it was not feasible to fit the model to all 203 subjects. To simplify our analysis, we assumed that distributions of tracts in the human brain were similar. With this assumption, we averaged the streamline count matrices ***S***_*i*_ (*i* = 1, …, 203) across subjects to obtain a single structural data matrix ***S*** and applied the first step of our pipeline to this resulting matrix, with the same specifications we used in our simulation study. In doing so, we aimed to study common features of the structural connectivity network of human subjects.

**Figure 10.**
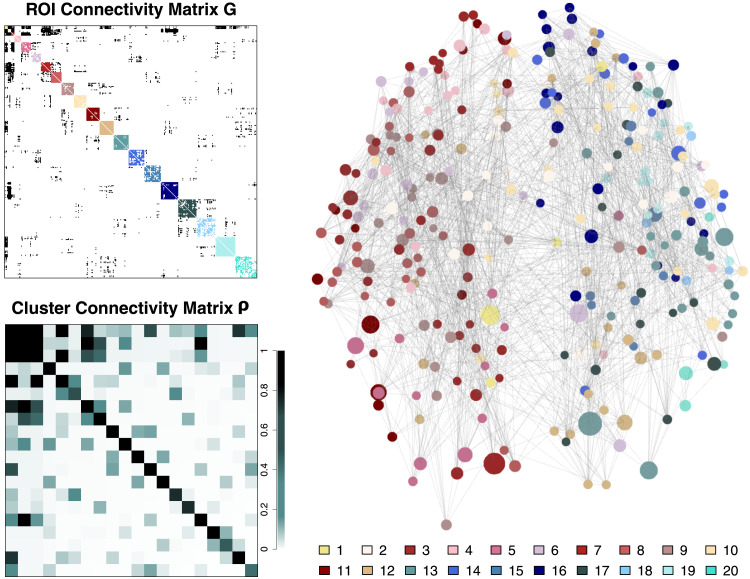
Empirical result: ROI connectivity matrix ***G*** and cluster connectivity matrix ***ρ***. The top-left panel shows the estimated ROI connectivity matrix ***G***. Within-cluster edges are color-coded to distinguish clusters, and between-cluster edges are colored black. White-coded cells indicate the absence of edges between ROIs. The bottom-left panel shows the estimated cluster connectivity matrix ***ρ***. Intra- and intercluster connectivity values are color-coded according to the legend on the right-hand side of the panel. The right panel shows the network image of ***G***. Nodes represent ROIs and gray lines represent edges between nodes. Node sizes are proportional to the surface areas of the ROIs. ROI nodes are color-coded according to the legend on the bottom-right side (as done for the top-left panel).

We used the clustering method of our pipeline to analyze the aggregated streamline count data ***S*** using the same procedure as in the simulation studies above. The resulting structure consisted of 20 clusters with varying sizes (4–26 ROIs). The cluster assignment result can be found in our online Supporting Information, which also includes the Gordon parcellation IDs, MNI centroid coordinates, surface areas, and cluster memberships of the ROIs examined. Figure 10 shows the clustered structural network of the ROIs in the Gordon parcellation and the connectivity profiles of the estimated clusters. The top-left panel shows the estimated ROI connectivity matrix ***G***. Within-cluster edges are color-coded with different colors to distinguish clusters (see the legend on the bottom-right side of the figure) and between-cluster edges are colored black. White-coded cells indicate the absence of edges between ROIs. The bottom-left panel shows the estimated cluster connectivity matrix ***ρ***. Intra- and intercluster connectivity values are color-coded according to the legend on the right-hand side of the panel. The clusters in the bottom-left panel are sorted by their size, and the ROIs in the top-left panel are also sorted accordingly. The right panel shows the network image of ***G***. Nodes represent ROIs and gray lines represent edges between nodes. Node sizes are proportional to the surface areas of the ROIs. ROI nodes are color-coded according to the legend on the bottom-right side (as done for the top-left panel). A 3D rotatable version of the network image is available in our Supporting Information.

The obtained clusters and their within- and between-cluster connectivity have some similarities and differences from the earlier result obtained by Hinne et al. (2015, their Figure 2). Within-cluster connectivity values varied in the range of [0.416, 0.993] (mean = 0.843 and *SD* = 0.163). Clusters 18 and 20 have relatively low within-cluster connectivity of 0.467 and 0.416, and clusters 6, 14, and 15 have mild levels of within-cluster connectivity (0.662, 0.740, and 0.740, respectively). These clusters also generally do not have large intercluster connections, but some edges to some clusters (cluster 6 to clusters 5 and 14, cluster 20 to clusters 1 and 12, etc.). The other clusters have rather strong within-cluster connectivity with the values varying in the range of [0.845, 0.993]. In particular, clusters 12 and 8 have within-cluster connectivity of 0.993 and 0.988, respectively. Cluster 12 has particularly many edges to cluster 1 (between-cluster connectivity of 0.643), and cluster 8 to clusters 2 (0.648) and 3 (0.426).

Clusters 1, 2, and 3 are small and densely connected. They have four ROIs each and so there can be six within-cluster edges. The model estimated that all six within-cluster edges exist for all these three clusters and the corresponding within-cluster connectivity estimates were 0.875 (not 1, due to *α* = 1 and *β* = 1 in the prior distribution). These clusters also have many intercluster connections as shown by the estimated cluster connection matrix ***ρ*** in Figure 10.

**Figure 11.**
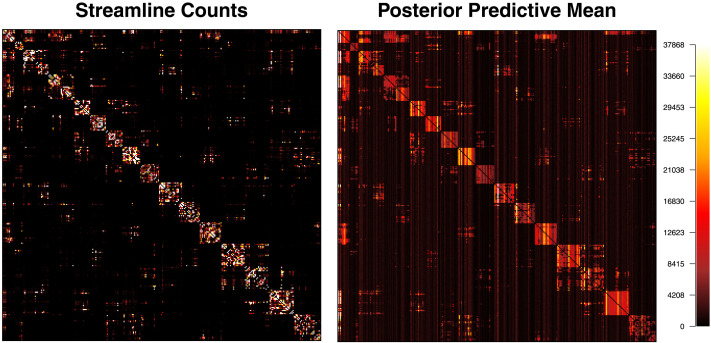
Absolute model fit. The left panel shows the streamline count data matrix and the right shows a posterior predictive distribution generated by simulating the clustering model 1,000 times. Both matrices are color-coded according to the legend on the far-right side.

To examine the absolute model fit, we conducted a posterior predictive check to examine whether the model can reproduce the connectivity patterns implied in the streamline count data matrix. To do so, we generated 1,000 posterior predictive samples of the streamline count matrix ***S*** and computed their posterior mean. In Figure 11, the left panel shows the data matrix and the right panel shows the predicted streamline count matrix. Both matrices are color-coded according to the legend on the far-right side. Because there were extremely large streamline counts between some ROIs in the data, we colored streamline counts more than 37,869 (99.9% quantile of the predicted streamline counts) with white. The result shows that the model can recover the connectivity pattern of the ROIs observed from our data. There is a difference in that the model predicts more intermediate numbers of edges (more reddish and more yellowish cells), whereas the data streamline counts have values that are more extreme.

### Functional Data Analysis

The next step in the pipeline was to fit FA models to the functional data with the information on the structurally connected clusters we obtained from our first step. Our functional data were collected from *I* = 203 subjects with *C* = 9 conditions (eight tasks and the resting state), producing 1,827 separate datasets. Because functional connectivity can largely differ by subject and task, we did not aggregate any data points. Instead, we fit FA models to all the datasets and used the outcomes for follow-up analyses, but for some outcome figures (e.g., factor loading and correlation matrices) we randomly selected five subjects (except for those who repeated the experiment) and provide the corresponding results for illustration of Step 2 of the pipeline. We fitted both EFA and CFA to each of these datasets. One complication is that the number of time points in the functional data ranged from 138 to 222 in the nine examined conditions (mean = 166.8 and standard deviation = 29.4), which is much fewer than the number of ROIs (*P* = 305). As a result, our functional data have a non-positive-definite correlation matrix for each subject-condition pair. To address this, we added a small positive value (0.01) to the diagonal entries of the correlation matrix and convert the outcome back to a correlation matrix. This produced a positive-definite correlation matrix with the original correlation structure and values maintained. By fitting FA models to the resulting correlation matrix, we aimed to illustrate the second step of the proposed pipeline and study functional coactivations of 305 ROIs with the 20 underlying structural clusters.

Because of large correlations and noise in data correlation matrices, converged CFA solutions had non-positive-definite factor correlation matrix (**Φ**) estimates with very large functional correlations between many factors (clusters). This implies that the loading structure informed by the obtained physical brain clusters was too restrictive to capture the underlying correlation structure of functional coactivation of ROIs, and some cross loadings (i.e., direct functional edges between ROIs in different clusters) were necessary to appropriately explain empirical patterns. Therefore, we chose to utilize EFA for a further investigation of the functional network (according to our criteria introduced in the section Choosing between EFA and CFA), as it has considerable flexibility in estimating the factor loading structure and capturing the observed correlation structure.

**Figure 12.**
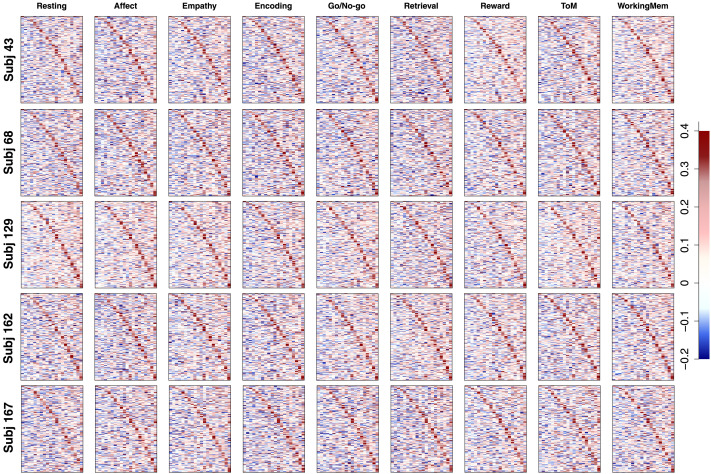
Empirical result: Factor loading matrices of functional connectivity. Each row corresponds to a different randomly selected subject, whereas the columns correspond to a different task. In each panel, the estimated factor loading matrix is shown, where the elements are color-coded according to the key on the right-hand side.

**Figure 13.**
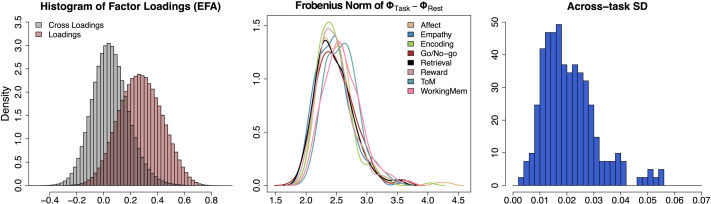
Empirical result: Factor loadings, cross loadings, and individual differences in factor correlations. The left panel shows the histograms of loadings (red) and cross loadings (gray), with the y-axis representing the densities. The middle panel shows the distribution of ∥**Φ**_*Task*_ − **Φ**_*Rest*_∥_*F*_ across subjects, but separately by task (see the legend on the top-right side), which represents individual differences in functional coactivations by task. The right panel shows the histogram of the subject-wise standard deviations (SD) in mean correlation values across tasks, quantifying within-subject across-task differences. **Φ**_*Task*_: Functional factor correlation matrix in a task. **Φ**_*Rest*_: Functional factor correlation matrix in the resting-state. ∥***A***∥_*F*_: Frobenius norm of a matrix ***A***.

Figure 12 shows the estimated factor loading matrices for five randomly selected subjects. Each row corresponds to a subject and each column corresponds to a different task. Because the loading structure was informed by the target matrix (clusters from the structural data analysis), the resulting factor loading matrices are generally similar. Although several of the estimated cross loadings had nonzero values to capture some proportions of data correlations, most loadings were much smaller. The left panel in Figure 13 shows the histograms of loadings (red) and cross loadings (gray) from all 203 subjects and nine conditions. Because there are more cross loadings than loadings (305 loadings and 305 · 20 − 305 = 5,795 cross loadings for each subject-condition pair), the height of the histograms shows the densities instead of frequencies. The histograms show that loadings generally have higher values (mean = 0.273, standard deviation = 0.159) than cross loadings that are centered at near 0 (mean = 0.051, standard deviation = 0.137) as the estimated structural network informed. However, some cross loadings have fairly large values; about 6.5% of the cross loadings have absolute values larger than the mean of loadings. The distribution of cross loadings show that they capture some functional coactivations, which implies that ROIs in different clusters may have some direct functional connections despite the absence of the corresponding structural edges.

**Figure 14.**
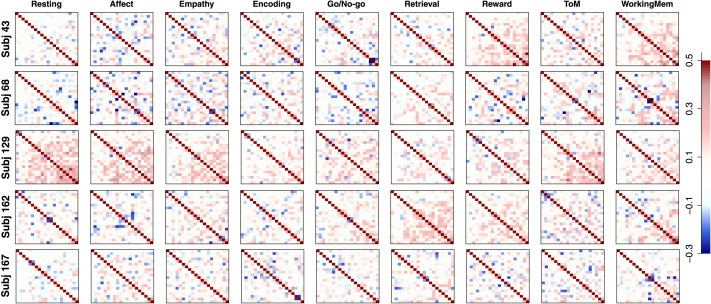
Empirical result: Factor correlation matrices of functional connectivity. Each row corresponds to a different randomly selected subject, whereas the columns correspond to a different task. In each panel, the estimated factor correlation matrix is shown, where the elements are color-coded according to the key on the right-hand side.

Figure 14 shows the estimated factor correlation matrices. As in Figure 12, the rows correspond to subjects and the columns correspond to tasks. All elements of the matrices are color-coded according to the legend on the far-right side. Because most of the correlation values were between −0.5 and 0.5, all elements with values higher than 0.5 (or lower than −0.5) were thresholded for visual clarity. The figure shows large heterogeneity in the factor correlations across subjects and tasks. For example, Subject 129 showed generally strong correlations while Subject 167 showed much weaker correlations across all nine conditions. Also, Subject 129 showed particularly strong correlations during the resting state and the Affect task, while the functional correlations were weaker in the Encoding and Retrieval tasks. To further investigate differences in factor correlations across subjects and across tasks, the middle and the right panels in Figure 13 quantify and visualize these differences. First, we measured the differences in factor correlations between a task and the resting state for each subject and each task so that we can study the changes in functional coactivations with the resting state as a general reference. The differences were measured as ∥**Φ**_*Task*_ − **Φ**_*Rest*_∥_*F*_, where **Φ**_*Task*_ and **Φ**_*Rest*_ are the estimated factor correlation matrices for one task and for a resting state, respectively (per subject-task pair, although subscripts for subjects and tasks are omitted), and ∥***A***∥_*F*_ represents the Frobenius norm of a matrix ***A***. The middle panel in Figure 13 shows the distribution of the Frobenius norm across subjects, but separately by task. For each task, the spread of the corresponding distribution shows the individual differences in functional coactivations. Comparing the distributions across tasks, generally there seem to be no noticeable differences between tasks. However, across-task differences can better be evaluated at the single-subject level because across-subject differences can hide heterogeneity due to task differences. For this, we first computed the mean correlation values per subject-task pair and then the standard deviation of these mean values over tasks, but separately by subjects. Thus, this standard deviation for a single subject represents across-task differences in mean functional coactivations of the subject. The right panel of Figure 13 shows the histogram of these standard deviation values. The histogram shows that some subjects have a small standard deviation across tasks while others have much larger standard deviations. With this result, we concluded that the factor correlation matrix estimated from the FA model, informed by the obtained structural connectivity network, shows changes in intercluster brain activation over different tasks. The same result also shows that there are large individual differences in how brain clusters are functionally connected.

**Figure 15.**
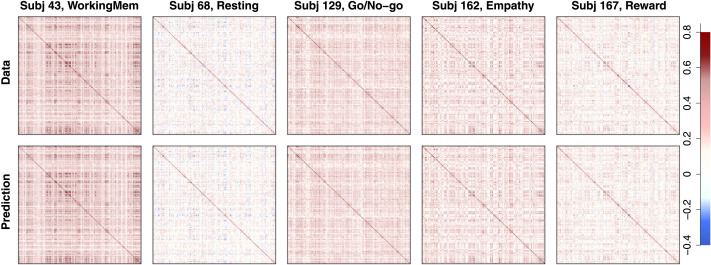
Absolute model fit. Comparison between the data correlation matrices (top row) and their corresponding implied (reproduced) correlation matrices (bottom row). Five pairs of subjects and conditions are selected for an illustration (columns).

**Figure 16.**
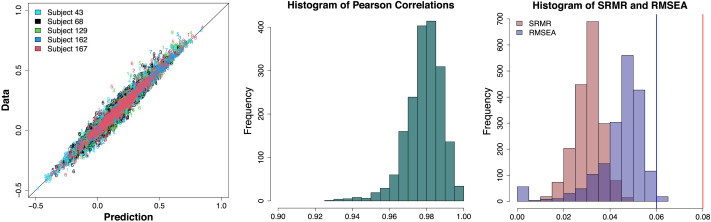
Absolute model fit. The left panel shows the scatterplot of data and predicted correlation values for 9 tasks and randomly selected 5 subjects. The lower triangular elements of the implied (reproduced) correlation matrix were plotted on the x-axis against the corresponding data correlation values. Correlations from different subjects were color-coded according to the legend on the top left and numbers 1–9 indicate different tasks (see below). The middle panel shows the histogram of the Pearson correlations between lower triangular elements of the data correlation matrix and their corresponding predictions across all 203 subjects and 9 tasks. The right panel shows the histograms of the root mean squared residuals (SRMR; red) and the root mean squared error of approximation (RMSEA; blue) across all 203 subjects and 9 tasks. The red and blue vertical lines indicate the upper bound criteria for a good fit for SRMR and RMSEA, respectively. Numbers in the left panel (tasks): 1 = Resting state, 2 = Affect, 3 = Empathy, 4 = Encoding, 5 = Go No-go, 6 = Retrieval, 7 = Reward, 8 = Theory of mind, 9 = Working memory.

Last, we examined the model’s absolute fit to functional data by comparing the data correlations matrices and their corresponding implied (reproduced) correlation matrices. Figure 15 shows the empirical data in the top row and the model prediction in the bottom row for some selected pairs of subjects and tasks (columns). Because there were 1,827 correlation matrices, we again employed those randomly selected subjects in Figure 12 and Figure 14 and chose one task for each subject for an illustration. Then, the correlation matrices for selected pairs of subjects and conditions and the corresponding model predictions were used to generate Figure 15. The Pearson correlations of lower triangular elements of the matrices between data and prediction were 0.984, 0.955, 0.975, 0.981, and 0.976 for the five pairs of subjects and conditions presented, and there were no noticeable biases in the predictions. Model fits were further examined with SRMR (Jöreskog & Sörbom, 1981) and RMSEA (Steiger & Lind, 1980). SRMR was smaller than 0.044 and RMSEA was smaller than 0.059 for all pairs of subjects and tasks in Figure 15, satisfying the upper bound for a good fit of 0.08 for SRMR and 0.06 for RMSEA (Hu & Bentler, 1998, 1999). Figure 16 provides a more thorough investigation of the absolute model fits. The left panel shows the scatterplot of data and predicted correlation values for nine tasks and randomly selected five subjects. The lower triangular elements of the implied (reproduced) correlation matrix were plotted on the x-axis against the corresponding data correlation values. Because each correlation matrix has 304 · 305/2 = 46,360 lower triangular elements, 500 elements were randomly sampled and plotted for each matrix (22,500 points in the plot). Correlations from different subjects were color-coded according to the legend on the top left and numbers 1–9 indicate different tasks (see the figure caption). The middle panel shows the histogram of the Pearson correlations between lower triangular elements of the data correlation matrix and their corresponding predictions across all 203 subjects and nine tasks. The mean and standard deviation of correlation values were 0.979 and 0.010, respectively, and the lowest correlation value was 0.927. The right panel shows the histograms of SRMR (red) and RMSEA (blue) across all 203 subjects and nine tasks. The red and blue vertical lines indicate the upper bound criteria for a good fit for SRMR and RMSEA, respectively. The maximum SRMR was 0.047 and so model fits for all subjects and tasks satisfied the criterion. There were 18 out of 1,827 cases with RMSEA larger than the criterion value of 0.06, but the maximum value of 0.063 did not deviate too much from the criterion. In general, Figure 15 and Figure 16 show that the absolute model fits were good. Because the EFA specifies such sparse constraint from the structural result, it is perhaps unsurprising that the model is able to fit the data well because of its flexibility. However, the obtained factor correlations can be interpreted as a lower bound of the underlying intercluster functional connectivity, as our simulation study suggested.

### Linking Functional Activation to Behavior

The proposed pipeline aims to constrain functional data analysis by structural clusters and identify individual and task-dependent differences in cluster-wise functional coactivations. The next question is what these differences imply for behavioral data analysis. For example, functional dependency of some brain clusters can be predictive of accuracy in cognitive tasks and it can be either positively or negatively related to the performance. Predicting behavior from neural data is not simple because relations between functional and behavioral measures can be complicated and simple methods such as correlations may not be an appropriate way to quantify underlying associations of those measures. A formal mathematical model can be built to better account for behavior and its related cognitive processes, but different tasks and different types of behavioral measures may require different mathematical models. For example, the standard diffusion decision model (Ratcliff, 1978; Ratcliff & McKoon, 2008) can be applied to response accuracy and response time of binary decision-making tasks (e.g., Encoding, Retrieval, and Working Memory tasks in our empirical application), but it would need to be modified to fit data from other tasks such as the Go/No-go task and the Reward task.

Because completing a functional-behavioral link by building a mathematical model is beyond the scope of this paper, we examined the potential of this link by simply regressing behavioral measures by functional coactivations. To this end, we utilized the factor correlation values obtained in Step 2 of the pipeline as predictors. Also, we used task responses as behavioral measures, which is task accuracy for most of the tasks but averaged momentary rewards for the Reward task and averaged 4-point Likert responses for the Encoding task of episodic memory. Because we have 18 · 19/2 = 190 factor correlations, predicting behavioral measures with all of these correlation values does not provide meaningful and reliable results. Instead, we implemented **Lasso regression** (least absolute shrinkage and selection operator; Friedman, Hastie, & Tibshirani, 2010; Tibshirani, 1996) to detect across-cluster functional coactivations that are predictive of behavioral performance. The Lasso regression penalizes complex models with a large number of predictors by the penalty defined as the *l*_1_-norm of regression coefficients. Importantly, the Lasso regression can remove some unimportant predictors by shrinking their regression coefficients to 0 while estimating the model (i.e., simultaneous model estimation and selection). In our application, we expect that the Lasso penalty will reduce many of the factor correlations, allowing only the most important functional coactivations to survive. At the same time, however, it is hard to expect that this simple regression-based approach can discover all significant functional-behavioral relations.

**Figure 17.**
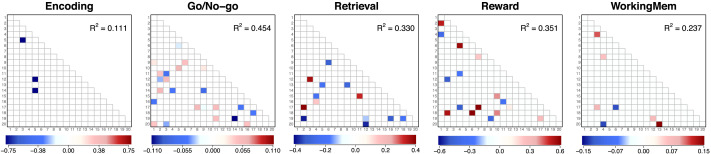
Functional-behavioral analysis. Each panel shows the lower diagonal entries of the functional factor correlation matrix **Φ** for the task shown on the top of the panel. The factor (cluster) numbers are shown on the rows and columns of each panel. The colored cells indicate the factor correlations that significantly predict performance measures. Those cells are color-coded according to the legend on the bottom of each panel. On the top-right side of each panel, the R-squared for the regression model only with the selected predictors is shown.

We fit the Lasso regression model to all eight tasks (except for the resting-state condition in which there is no performance measure) and found some significant predictors for five tasks: Encoding, Go/No-go, Retrieval, Reward, and Working Memory tasks. Figure 17 summarizes our results. Each panel shows the lower diagonal entries of the functional factor correlation matrix **Φ** for the task shown on the top of the panel. The factor (cluster) numbers are shown on the rows and columns of each panel. The colored cells indicate the factor correlations that significantly predict performance measures. Those cells are color-coded according to the legend on the bottom of each panel. On the top-right side of each panel, the R-squared for the regression model only with the selected predictors is shown to present how much variance in performance measures can be explained by the remaining predictors. For example, for the Working memory task, factor correlations of cluster pairs (3, 4), (3, 17), (4, 8), (4, 20), (6, 17), (12, 19), and (13, 20) were significant predictors for response accuracy of subjects. Higher functional coactivations of these cluster pairs were positively associated with response accuracy, except for the (4, 20) and (6, 17) pairs, which had negative associations. These factor correlations accounted for about 23.7% of the variances in response accuracy.

Across the five tasks, about 11.1%–45.4% of the variances were explained by significant functional between-cluster coactivations. These explanations are promising given that they are the results based on simple correlations between performance measures and functional factor correlations. We expect that mathematical models can be built to disentangle the underlying associations between functional coactivations and behavior and provide a better model-based account for a functional-behavioral link. This modeling can be further strengthened by incorporating other measures such as response time (behavior), factor loading, and factor scores (functional). For the other three tasks (Affect, Empathy, and Theory of Mind), we would also expect that there could be some kind of associations that can be discovered by behavioral models with functional coactivations combined, despite that the regression-based approach we utilized failed to reveal meaningful relations.

## DISCUSSION AND CONCLUSION

In this article, we proposed a two-step pipeline to analyze structural and functional brain connectivity data. The pipeline aimed to explore the undiscovered physical clusters of brain areas without any prior knowledge (e.g., number of clusters), and then map the obtained structural clusters onto functional connectivity data to study how clusters functionally interact during cognitive tasks. The first step of the pipeline employed a nonparametric Bayesian clustering method built based on the Chinese restaurant process, which defines a distribution for cluster memberships of ROIs with a potentially infinite number of clusters. The second step used a FA model to define latent factors of functional activation of brain areas by structural clusters and to capture their interaction by factor correlations.

We applied the proposed pipeline to data from Gaut et al. (2019) that included streamline counts of ROIs defined by the Gordon parcellation and fMRI datasets collected during resting state and eight cognitive tasks. The first step produced an estimated structural network with 20 physical clusters of brain areas. The clusters vary in their size (4–26 ROIs) and most of them have strong within-cluster connectivity, while they have substantial heterogeneity in the between-cluster connectivity. In the second step, we applied the EFA model to functional data for which the factor loading structure was informed by the clustered network of structural data we obtained in the first step. We found a strong consistency in the factor loading matrices across subjects and conditions, while there were large differences in the factor correlations. This result showed that we can explore individual differences in functional connectivity networks, and the degree to which these functional networks change across tasks within a single subject.

The novelty of the proposed pipeline comes from our attempt to (a) identify functional brain clusters (factors) informed by the structural clusters obtained without any a priori knowledge, and (b) investigate task-dependent coactivations of the functional brain clusters. In fact, neither component of the model in our pipeline is new in terms of its applications to brain connectivity analyses. Earlier clustering methods such as the k-means method (Hartigan & Wong, 1979) and the Infomap algorithm (Rosvall & Bergstrom, 2008) have been applied to brain connectivity data (Allen et al., 2014; Anwander et al., 2007; Hansen et al., 2015; Hinne et al., 2015; Mars et al., 2011). However, these methods require the number of clusters specified a priori, which is unknown. Unsupervised clustering methods (Knösche & Tittgemeyer, 2011), including the IRM (Kemp et al., 2006; Xu et al., 2007) we used here, are free from this limitation as they can simultaneously estimate the number of clusters and cluster assignments. IRM also has been applied to structural (Hinne et al., 2015) and functional connectivity data (Andersen et al., 2014; Mørup et al., 2010), but the resulting clustered network has not been incorporated into the further analysis to study the other connectivity.

Applications of the FA model to brain imaging data are also not new. For example, exploratory models with latent variables have been applied to resting-state fMRI (James et al., 2009) and EEG (Scharf & Nestler, 2018; Tucker & Roth, 1984). As one example, van Kesteren and Kievit (2021) proposed an EFA model with a structured residual matrix and applied it to structural and functional connectivity data. However, earlier latent variable approaches require the number of factors specified a priori and estimate associations between brain areas and factors (i.e., loadings). In this case, the number of factors and factor loading structure should be determined solely by the information from the data being analyzed, and thus, interpretations of the extracted factors remain arbitrary particularly when functional connectivity data were analyzed. Turner, Wang, and Merkle (2017) and Kang et al. (2022) solved this issue by jointly modeling behavioral and brain data in which cognitive components of behavioral decision processes define the latent variables and the association between these latent variables and ROIs in the brain were investigated. Our pipeline provides an alternative solution that constrains the functional latent variables by the physical clusters of brain areas. This approach allows one to study the changes in functional coactivations of clusters across different cognitive tasks, changes that are relative to a common structural basis. An alternative way to determine the number of factors without specifying it a priori is to implement a nonparametric FA model (Gershman & Blei, 2012; Knowles & Ghahramani, 2011). As the IRM applied to structural data, this nonparametric model can determine the number of factors while estimating the factor loading matrix. A complication is that the resulting factor loading structure is largely determined by data and it remains to find a plausible interpretation of extracted factors. When applied to psychometric tests or personality inventories, obtained factors can be interpreted based on item contents (e.g., item sentences of personality inventories, required skills to solve items in mathematical tests). However, it is hard to find such interpretations of factors with ROI activations. Our suggested approach can avoid this issue as factors are connected to structural brain clusters.

Despite having verified that the pipeline works well in several simulation studies, there are still several issues that remain to be further investigated. Most importantly, potential interpretations and uses of the obtained across-subject and across-task differences in functional connectivity (measured as factor correlations) should be found. One possibility is to see whether behavioral measures such as subjects’ performance in the tasks examined can be predicted by the factor correlations. We also provided an example for this possibility with a preliminary analysis of a potential functional-behavioral link based on the Lasso regression.

However, associations of functional activity and human behavior are not as simple as correlations in functional and behavioral data, and a formal mathematical model should be built to disentangle complicated functional-behavioral links. A recent approach, called *model-based cognitive neuroscience*, relates aspects of neural measurements to parameters of a computational model. The advantage here is that computational models specify how behavioral variables manifest by connecting data to a set of latent variables. Because the mind is a latent construct, computational models specify the mind’s computations as a set of conditionally independent latent variables that can be estimated from data, rather than as a direct transformation of either neural or behavioral data (see Turner, Forstmann, Love, Palmeri, & Maanen, 2017, for discussion). One especially relevant application of model-based cognitive neuroscience for our purposes is the neural drift diffusion model (NDDM; Turner, van Maanen, & Forstmann, 2015), which specifies a link between neural states for a set of ROIs to the parameters of the diffusion decision model (DDM; Ratcliff, 1978). The DDM, and by extension the NDDM, uses sets of parameters to describe the joint distribution of choice and response time, and these parameters have been related to many cognitive abilities such as the intelligence quotient other intelligence-related tasks (Frischkorn & Schubert, 2018; Kang, De Boeck, & Partchev, 2022; Lerche et al., 2020; McKoon & Ratcliff, 2012; Ratcliff, Schmiedek, & McKoon, 2008; Ratcliff, Thapar, & McKoon, 2009, 2011). The NDDM extends the DDM by simply specifying a functional form that connects the functional coactivation of brain data to the parameters of the model. This extension was shown to be effective in that it increased the accuracy of predictions about response time and probability of errors in a perceptual decision-making task. The NDDM was further refined to specify how functional coactivation matrices may be equally well described by a factor analytic structure (Turner, Wang, & Merkle, 2017), which can be effectively regularized to arrive at parsimonious linking functions (Kang et al., 2022). Importantly, the factor structure of functional data in the NDDM, which is decomposed by the behavioral parameters, can be related to another factor structure informed by the structural connectivity network. This can be done by simultaneously analyzing functional and behavioral data with a confirmatory factor structure, which is spanned by behavioral factors (such as the DDM parameters) and structural factors (such as structural clusters as in our pipeline). This provides a promising future extension of the pipeline proposed in this paper.

Another issue is the two-step specification of the models. In the current two-step pipeline, the structural connectivity estimates from the first step were considered the ground truth in the second step. This approach is limited in that the uncertainty in the first step estimates is ignored when forming estimates in the second step. Clearly, such a shortcoming can be problematic when the result from the first step has some unexpected errors. Although our simulation study showed robust recovery of the underlying structural network in several different scenarios, there unfortunately are no guarantees when applying the IRM to real data. An ideal approach would be to build a single model that would allow not only the structural data to constrain the functional data, but also the functional data to inform the structural data. For example, Hinne et al. (2013) simultaneously analyzed structural and functional data by combining the DCM with the G-Wishart distribution (Roverato, 2002) as a prior distribution for a precision matrix of functional data. The G-Wishart distribution provides a way to constrain functional connectivity based on the information from structural data, but this approach did not implement a clustering prior for structural connectivity and constraints were imposed on the edges between ROIs. If it were determined that a clustered network of activity was necessary during a particular subset of cognitive tasks, this information could be used to expand the network of the structural connectivity result. Unfortunately, a major complication is that the shape of the factor loading matrix (i.e., which loading is active and inactive) and the number of factors (i.e., the column size of the loading matrix) in the functional connectivity analysis would continually change over iterations as a function of the clustered structural network obtained on that iteration. One way to partially deal with this issue is to introduce a decomposition of the factor loading matrix, **Λ** = ***Z*** ∘ ***L***, where ∘ denotes the element-wise multiplication (i.e., the Hadamard product) of matrices and ***L*** is the factor loading matrix with all loadings allowed to have nonzero values. With this decomposition, only the active loadings informed by the structural cluster information in ***Z*** contribute to the model likelihood. Although this is a direct extension of the proposed pipeline, it is inherently a mixture model of infinitely many possible loading structures, which makes it difficult to find a solution. Future work will need to produce an efficient algorithm to find the optimal solution if a single, unified nonparametric model of structural and functional connectivity is to be established.

## SUPPORTING INFORMATION

Supporting information for this article is available at https://doi.org/10.1162/netn_a_00242.

## AUTHOR CONTRIBUTIONS

Inhan Kang: Conceptualization; Formal analysis; Investigation; Methodology; Validation; Visualization; Writing – original draft; Writing – review & editing. Matthew Galdo: Data curation; Investigation; Writing – review & editing. Brandon Turner: Conceptualization; Data curation; Funding acquisition; Investigation; Supervision; Visualization; Writing – original draft; Writing – review & editing.

## FUNDING INFORMATION

Brandon Turner, National Science Foundation (https://dx.doi.org/10.13039/501100008982), Award ID: CAREER.

## Supplementary Material



## TECHNICAL TERMS

Factor analysis: Statistical analysis that explains a correlation structure of observed variables as a function of common underlying latent variables called factors.
Infinite relational model: A nonparametric Bayesian model that decomposes a binary connectivity matrix into systematic clusters of variables.
Dirichlet compound multinomial distribution: Discrete multivariate distribution in which a multinomial distribution for nonnegative integer observations and a Dirichlet distribution for their probability vector are combined.
Chinese restaurant process (CRP): A stochastic process that is analogous to customers (variables) and tables (clusters of variables) in a Chinese restaurant.
Community-based clusters: Brain clusters in which ROIs are densely connected within the cluster but are less likely to be connected to ROIs in different clusters.
Profile-based clusters: Brain clusters in which ROIs within the cluster are not necessarily highly interconnected but tend to have similar connectivity profiles.
Maximum a posteriori: A point estimate of a parameter used in Bayesian inference, which is equal to the mode of the posterior distribution.
Standardized root mean squared residuals: Model fit index used in factor analysis, defined as the square root of the mean squared residuals between observed and predicted correlations.
Root mean squared error of approximation: Model fit index used in factor analysis, defined based on the maximum likelihood objective function to be minimized to estimate model parameters.
Lasso regression (least absolute shrinkage and selection operator): Regression analysis method that penalizes models with many predictors by the *l*_1_ penalty (absolute values) of regression coefficients.
